# DNA Damage and DNA Damage Response in Chronic Myeloid Leukemia

**DOI:** 10.3390/ijms21041177

**Published:** 2020-02-11

**Authors:** Henning D. Popp, Vanessa Kohl, Nicole Naumann, Johanna Flach, Susanne Brendel, Helga Kleiner, Christel Weiss, Wolfgang Seifarth, Susanne Saussele, Wolf-Karsten Hofmann, Alice Fabarius

**Affiliations:** 1 Department of Hematology and Oncology, Medical Faculty Mannheim, Heidelberg University, 68167 Mannheim, Germany; Vanessa.Kohl@medma.uni-heidelberg.de (V.K.); Nicole.Naumann@medma.uni-heidelberg.de (N.N.); Johanna.Flach@medma.uni-heidelberg.de (J.F.); Susanne.Brendel@medma.uni-heidelberg.de (S.B.); Helga.Kleiner@medma.uni-heidelberg.de (H.K.); Wolfgang.Seifarth@medma.uni-heidelberg.de (W.S.); Susanne.Saussele@medma.uni-heidelberg.de (S.S.); w.k.hofmann@medma.uni-heidelberg.de (W.-K.H.); Alice.Fabarius@medma.uni-heidelberg.de (A.F.); 2 Department of Medical Statistics and Biomathematics, Medical Faculty Mannheim, Heidelberg University, 68167 Mannheim, Germany; Christel.Weiss@medma.uni-heidelberg.de

**Keywords:** chronic myeloid leukemia, genetic instability, DNA double-strand breaks, DNA damage response

## Abstract

DNA damage and alterations in the DNA damage response (DDR) are critical sources of genetic instability that might be involved in BCR-ABL1 kinase-mediated blastic transformation of chronic myeloid leukemia (CML). Here, increased DNA damage is detected by γH2AX foci analysis in peripheral blood mononuclear cells (PBMCs) of de novo untreated chronic phase (CP)-CML patients (*n* = 5; 2.5 γH2AX foci per PBMC ± 0.5) and blast phase (BP)-CML patients (*n* = 3; 4.4 γH2AX foci per PBMC ± 0.7) as well as CP-CML patients with loss of major molecular response (MMR) (*n* = 5; 1.8 γH2AX foci per PBMC ± 0.4) when compared to DNA damage in PBMC of healthy donors (*n* = 8; 1.0 γH2AX foci per PBMC ± 0.1) and CP-CML patients in deep molecular response or MMR (*n* = 26; 1.0 γH2AX foci per PBMC ± 0.1). Progressive activation of erroneous non-homologous end joining (NHEJ) repair mechanisms during blastic transformation in CML is indicated by abundant co-localization of γH2AX/53BP1 foci, while a decline of the DDR is suggested by defective expression of (p-)ATM and (p-)CHK2. In summary, our data provide evidence for the accumulation of DNA damage in the course of CML and suggest ongoing DNA damage, erroneous NHEJ repair mechanisms, and alterations in the DDR as critical mediators of blastic transformation in CML.

## 1. Introduction

Chronic myeloid leukemia (CML) is a myeloproliferative neoplasm characterized by increased proliferation of myeloid cells in the bone marrow and expansion of these cells in the peripheral blood [1]. The leukemic clone originates from a hematopoietic stem cell by acquisition of the chromosomal translocation t(9;22)(q34;q11) containing the *BCR-ABL1* fusion gene [1]. The natural course of untreated CML is characterized by an initial chronic phase (CP) that progresses to an accelerated phase (AP) and a terminal blast phase (BP) [2]. During this process, the constitutively activated BCR-ABL1 tyrosine kinase stimulates different oncogenic pathways (e.g., WNT, PI3K/AKT, JAK/STAT, Hedgehog signaling) [3], which drive malignant differentiation (e.g., proliferation, G2/M delay, cell survival) [4]. In addition, BCR-ABL1 kinase-mediated genetic instability (e.g., reactive oxygen species, replication stress, error-prone DNA repair, centrosomal dysfunction) presumably plays a critical role in the blastic transformation of CML [5,6,7,8]. 

Tyrosine kinase inhibitors (TKIs) have revolutionized CML therapy, and induce high rates of deep or major molecular responses (DMRs or MMRs) [9]. However, TKI failure may occur, for example, by acquired point mutations in the BCR-ABL1 tyrosine kinase domain, clonal evolution, or BCR-ABL1 independent pathways resulting in loss of MMR and disease progression [10].

DNA double-strand breaks (DSBs) are serious DNA lesions that may accumulate during the course of CML. In response to DSB, the histone variant H2AX is phosphorylated at Ser139 in a region of several megabase pairs around the DSB, resulting in the formation of discrete γH2AX foci in the nucleus that are detectable by immunofluorescence microscopy [11]. γH2AX recruits additional proteins engaged in chromatin remodeling, DNA repair, and signal transduction [12,13,14,15]. One of these proteins is 53BP1 [16], which promotes ATM-dependent checkpoint signaling, regulates DSB repair pathway choice, and tethers DNA ends during non-homologous end joining (NHEJ) [17,18]. Importantly, 53BP1 triggers the repair of DSBs by erroneous NHEJ and microhomology-mediated end joining (MMEJ), which may aggravate genetic instability [19,20,21]. In this study, γH2AX and 53BP1 foci were analyzed by immunofluorescence microscopy in peripheral blood mononuclear cells (PBMCs) of CML patients at different stages. 

The DNA damage response (DDR) is activated upon DNA damage and acts as an ”anti-cancer barrier” [22]. In this signaling network, the ATM-CHK2 and ATR-CHK1 axes promote the repair of DSB and single-strand breaks, respectively. In addition, TP53 may induce apoptosis if apoptotic factors overwhelm DNA repair factors [23,24,25]. However, the frequency of additional chromosomal aberrations (ACAs) is about 5% in CP-CML and increases to about 80% in BP-CML [26,27], making a strong argument for the occurrence of DDR defects in the course of CML. 

In summary, genetic instability is most likely involved in blastic transformation of CML. The goal of our project was to analyze mechanisms of genetic instability related to DNA damage, DSB repair, and DDR signaling in CML. For this purpose, immunofluorescence microscopy of γH2AX/53BP1 and Western blotting of (p-)ATM/(p-)CHK2 were applied in PBMC of CML patients at different disease stages in comparison to healthy controls. 

## 2. Results 

### 2.1. γH2AX Foci in PBMCs of Healthy Donors and CML Patients

**Figure 1:**
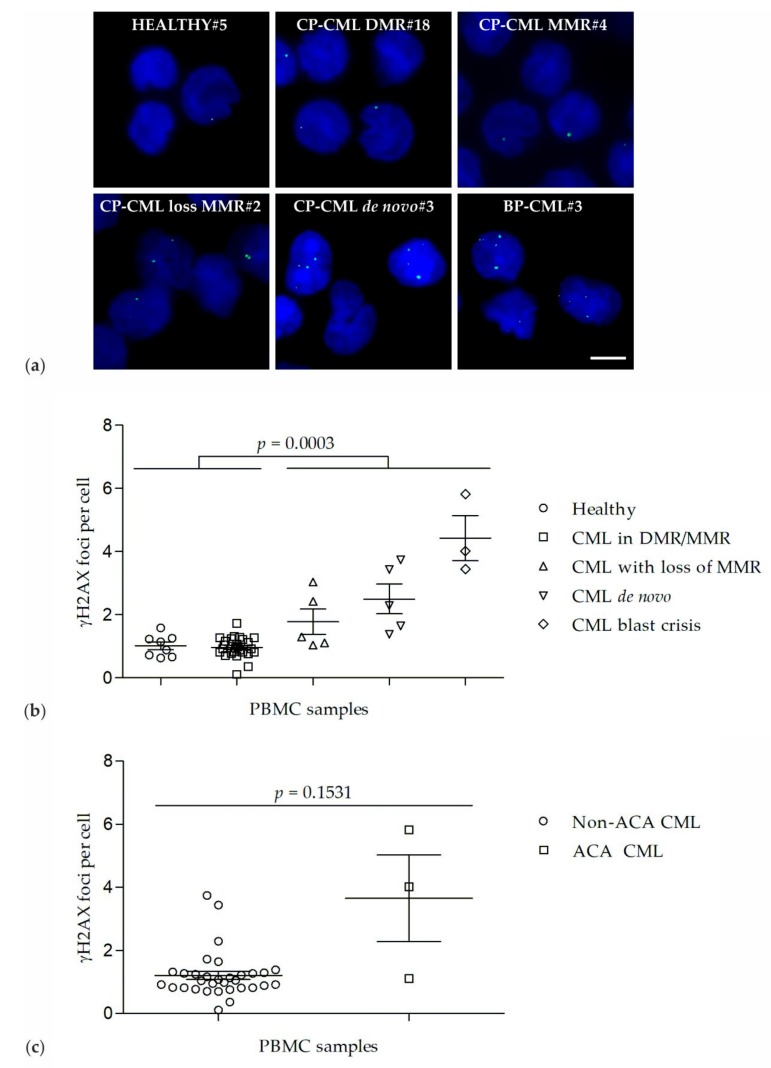
γH2AX foci in peripheral blood mononuclear cells (PBMC) of healthy donors and CML patients. (**a**) Representative recordings of γH2AX foci (green, Alexa 488) in PBMC nuclei (blue, DAPI) of a healthy donor (HEALTHY#5), a CP-CML patient with a DMR (CP-CML DMR#18), a CP-CML patient with a MMR (CP-CML MMR#4), a CP-CML patient with a loss of MMR (CP-CML loss MMR#2), a de novo untreated CP-CML patient (CP-CML de novo#3), and a BP-CML patient (BP-CML#3). Scale bar = 5 µm. (**b**) γH2AX foci counts in PBMCs of healthy donors and in PBMCs of CML patients at different stages. (**c**) γH2AX foci counts in PBMCs of CML patients without additional chromosomal aberrations (Non-ACA CML) and in PBMCs of CML patients with additional chromosomal aberrations (ACA CML).

γH2AX foci were analyzed in PBMCs of healthy donors (*n* = 8, group 1), CP-CML patients in DMR or MMR (*n* = 22 + 4, group 2), CP-CML patients with loss of MMR (*n* = 5, group 3), de novo CP-CML patients (*n* = 5, group 4), and BP-CML patients (*n* = 3, group 5) (Table 1, Figure 1a,b). The number of γH2AX foci varied in each cell of a given sample. γH2AX foci levels were similar in PBMCs of healthy donors (1.0 γH2AX foci per PBMC ± 0.1) and in PBMCs of CP-CML patients in DMR/MMR (1.0 γH2AX foci per PBMC ± 0.1). Importantly, γH2AX foci levels were significantly increased (*p* = 0.0003) in PBMCs of de novo CP-CML patients (2.5 γH2AX foci per PBMC ± 0.5) and BP-CML patients (4.4 γH2AX foci per PBMC ± 0.7) as well as CP-CML patients with loss of MMR (1.8 γH2AX foci per PBMC ± 0.4) when compared to γH2AX foci levels in PBMC of healthy donors (1.0 γH2AX foci per PBMC ± 0.1) and CP-CML patients in DMR or MMR (1.0 γH2AX foci per PBMC ± 0.1) (Figure 1b). 

Furthermore, γH2AX foci levels were correlated with the detection of ACAs in CML (Figure 1c). γH2AX foci levels tended to be increased (*p* = 0.1531) in CML samples with ACAs (*n* = 3; 1 CP-CML sample with loss of MMR, 2 BP-CML samples) as compared to γH2AX foci levels in CML samples without ACAs (*n* = 32; 26 CP-CML samples in DMR/MMR, 1 CP-CML sample with loss of MMR and 5 de novo CP-CML samples). 

### 2.2. Co-Localization of γH2AX and 53BP1 Foci

**Figure 2:**
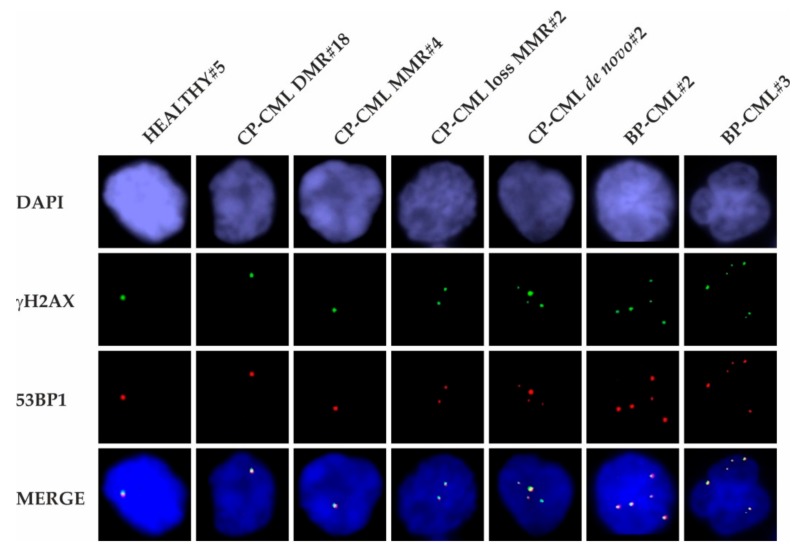
γH2AX and 53BP1 foci in PBMCs of healthy donors and CML patients. Representative recordings show co-localization of γH2AX foci (green, Alexa 488) and 53BP1 foci (red, Cy3) in PBMC nuclei (blue, DAPI) of a healthy donor (HEALTHY#5), a CP-CML patient with DMR (CP-CML DMR#18), a CP-CML patient with MMR (CP-CML MMR#4), a CP-CML patient with loss of MMR (CP-CML loss MMR#2), a de novo untreated CP-CML patient (CP-CML de novo#2), and two BP-CML patients (BP-CML#2 and BP-CML#3).

γH2AX and 53BP1 foci were analyzed in the PBMCs of healthy donors and all CML groups 2–5 (Figure 2). All samples showed co-localizing γH2AX/53BP1 foci in similar patterns. Notably, the number of γH2AX/53BP1 foci in PBMCs increased across the spectrum from CP-CML towards BP-CML patients, suggesting the promotion of erroneous NHEJ and MMEJ during blastic transformation.

### 2.3. DNA Damage Response

**Figure 3:**
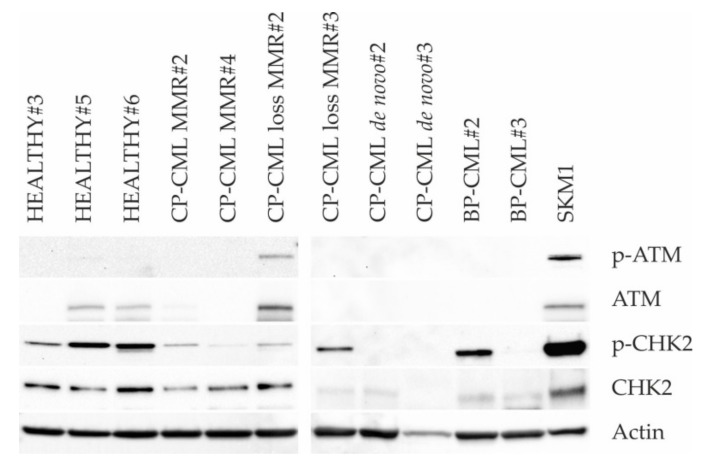
DNA damage response (DDR) in PBMCs of healthy donors and CML patients. Representative Western blots of (p-)ATM and (p-)CHK2 in PBMC of three healthy donors (HEALTHY#3, HEALTHY#5, AND HEALTHY#6), two CP-CML patients in MMR (CP-CML MMR#2 and CP-CML MMR#4), two CP-CML patients with loss of MMR (CP-CML loss MMR#2 and CP-CML loss MMR#3), two de novo untreated CP-CML patients (CP-CML de novo#2 and CP-CML de novo#3), and two BP-CML patients (BP-CML#2 and BP-CML#3). In PBMCs of healthy donors, CP-CML patients in MMR, and CP-CML patients with loss of MMR, no or minor expression of p-ATM and variable expression of p-CHK2 were evident. Notably, in PBMCs of de novo CP-CML patients and BP-CML patients, no expression of p-ATM and predominantly no expression of p-CHK2 were observed (except for strong expression of p-CHK2 in BP-CML#2). SKM-1 acute myeloid leukemia cells served as positive control.

Western blotting of (p-)ATM and (p-)CHK2 was performed in PBMCs of healthy donors and all CML groups 2–5 (Figure 3). PBMCs of healthy donors, CP-CML patients with DMR or MMR, and CP-CML patients with loss of MMR demonstrated no or minor expression of p-ATM and variable expression of p-CHK2, which was in accordance with the presence of low levels of DNA damage in these cells as evidenced by γH2AX foci analysis. However, PBMC of de novo CP-CML patients and BP-CML patients demonstrated no expression of p-ATM and predominantly no expression of p-CHK2 (apart from strong expression of p-CHK2 in BP-CML#2), which was in contrast to the presence of relatively high levels of DNA damage in these cells as evidenced by γH2AX foci analysis. The results suggest an alteration of the DDR in transformed CML cells.

## 3. Discussion

DSBs continuously occur in each genome [28] and are elementarily involved in the blastic transformation of CML, as evidenced by the formation of t(9;22)(q34;q11) in CP-CML and a high degree of ACA in BP-CML. For the detection of DSBs, the immunofluorescence microscopy of γH2AX and 53BP1 foci was performed in PBMCs of healthy donors, CP-CML patients in DMR/MMR, CP-CML patients with loss of MMR, de novo CP-CML patients, and BP-CML patients. Analyses showed a gain in DSB in the course from CP-CML towards BP-CML, indicating a biological continuum to accumulate DNA damage across different CML stages. Moreover, the intersection of γH2AX foci levels between different CML stages indicated that γH2AX foci were a biological marker, rather than a stage-specific marker. Further, γH2AX foci levels were evaluated in CML patient samples in relation to ACA. The ACA-bearing BP-CML samples demonstrated increased γH2AX foci levels as compared to the non-ACA bearing CP-CML samples. This demonstrates that γH2AX foci and ACA increase concordantly in the process of blastic transformation in CML.

γH2AX and 53BP1 foci were analyzed semi-quantitatively in PBMCs of all healthy donors and CML patients. Analyses revealed similar patterns of γH2AX and 53BP1 foci co-localization in all samples. Furthermore, co-localizing γH2AX/53BP1 foci increased in the course from CP-CML patients towards BP-CML patients. This observation suggests the promotion of erroneous NHEJ and MMEJ as critical mechanisms of blastic transformation in CML. Considering the contribution of erroneous NHEJ and particularly MMEJ to the formation of chromosomal aberrations such as deletions, translocations*,* inversions, and other complex rearrangements [29], this data might, at least in part, explain the occurrence of ACAs in BP-CML.

γH2AX and 53BP1 foci were evaluated in different CML patients at various stages. One might ask for follow-up data in same CML patients; however, none of the CML patients in DMR or MMR (group 2), who were treated by TKI or were closely monitored in a TKI–free setting, demonstrated loss of MMR during study. Further, CML patients with loss of MMR (group 3) were changed promptly to another TKI according to BCR/ABL kinase domain mutations and patient characteristics. These patients achieved DMR or MMR shortly again, except for one patient who took the TKI irregularly and still demonstrated loss of MMR. Although our study lacks follow-up data on γH2AX and 53BP1 foci in same CML patients, the potential increases in the long-term course in these patients may be similar to the numbers of γH2AX and 53BP1 foci in different CML patients at various stages.

Western blotting of (p-)ATM and (p-)CHK2 was performed in the PBMCs of healthy donors and CML patients. Minor activation of the DDR proteins was detected in the PBMCs of healthy donors, CP-CML patients in DMR or MMR, and CP-CML patients with loss of MMR, which was in accordance with the detection of low levels of DNA damage in these cells, as evidenced by γH2AX foci analysis. However, in the PBMCs of de novo CP-CML patients and BP-CML patients, almost no activation of the DDR proteins was observed, too, despite of the presence of relatively high levels of DNA damage in these cells, as evidenced by γH2AX foci analysis and the presence of ACAs. The absence of p-ATM and the predominant absence of p-CHK2 in the PBMCs of de novo CP-CML and BP-CML patients might be explained by missing or minor expression of ATM and CHK2, respectively. Further, there may also have been defective phosphorylation of ATM and CHK2, aggravating DDR defects. Overall, our results implicate alterations of the DDR in CML cells and correspond well with the known function of the DDR as an ”anti-cancer barrier” that becomes activated upon DNA damage in healthy cells and disarranged in cancer cells [22,30].

**Figure 4:**
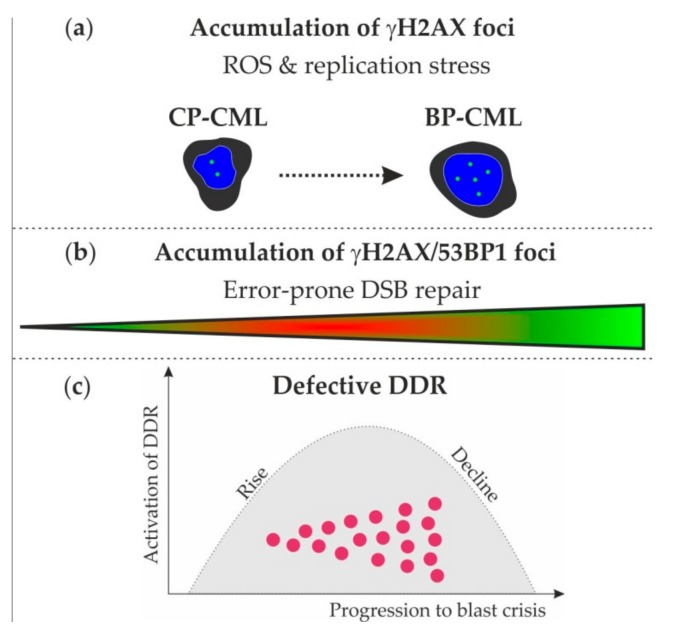
Model of genetic instability in CML. (**a**) Accumulation of γH2AX foci (green) in nuclei (blue) of CP-CML and BP-CML may increase with reactive oxygen species (ROS)- and replication-stress-induced DNA damage. (**b**) Accumulation of γH2AX/53BP1 foci suggests progressive activation of error-prone DNA double-strand break (DSB) repair in the course from CP-CML towards BP-CML. (**c**) After initial activation of the DDR in hematopoietic cells, the DDR might finally decline in transformed CP- and BP-CML cells.

Our data may generate hypotheses for the development of genetic instability in CML, in which γH2AX and 53BP1 foci accumulate with ongoing DNA damage by intrinsic sources (e.g., reactive oxygen species, replication-stress-induced DNA damage), erroneous DSB repair (e.g., NHEJ and MMEJ), and alterations of the DDR (e.g., sensing and signaling of DNA damage) (Figure 4). Importantly, genetic instability is a common feature in TKI-refractory CML. In consideration of the worse prognosis, the limited effectiveness of chemotherapy and the ineligibility of most of these patients for allogeneic bone marrow transplantation, novel treatment options are an unmet need at present. Here, DNA repair inhibitors (e.g., PARP or APE1 inhibitors) might be beneficial by inducing synthetic lethality in DNA repair defective TKI-refractory CML cells [31] and may constitute possible treatment options.

## 4. Materials and Methods

### 4.1. Blood Samples

This study was authorized by the Ethics Committee II of the Medical Faculty Mannheim of the Heidelberg University (2018-566N-MA). Written informed consent was given by all participants. Blood samples were collected from 8 healthy donors (3 males, 5 females, mean age: 50 years), 26 CP-CML patients in DMR or MMR (9 males, 17 females, mean age: 63 years), 5 CP-CML patients with loss of MMR (2 males, 3 females, mean age: 69 years), 5 de novo untreated CP-CML patients (1 male, 4 females, mean age: 47 years), and 3 BP-CML patients (1 male, 2 female, mean age: 59 years) (Table 1). Treatment with TKI included imatinib, nilotinib, dasatinib, and bosutinib, respectively. PBMCs were separated from whole-blood samples by Ficoll-Paque density gradient centrifugation (Miltenyi Biotec, Bergisch Gladbach, Germany).

### 4.2. Cytology, Cytogenetics, and Molecular Analyses

Peripheral blood smears were stained by May-Gruenwald-Giemsa [32]. Fluorescence in situ hybridization (FISH) of *BCR-ABL1*-rearrangement was performed with probes for the detection of t(9;22)(q34;q11) [33]. ACAs were determined by conventional cytogenetics of G-banded chromosomes [34]. The molecular response (MR) of CML was assessed by international scale (IS)-standardized polymerase chain reaction (PCR) of *BCR-ABL1* in all CML samples [35]. For the quantitative real-time PCR reaction (qRT-PCR) of *BCR-ABL1* fusion gene and *GUSB* control gene, a TaqMan detection system (TaqMan 7500 Fast Real-Time PCR System, Thermo Fisher Scientific, Waltham, US) was used for quantification of e13a2/e14a2 (b2a2/b3a2) transcripts and a LightCycler detection system (Roche Applied Science, Penzberg, Germany) for quantification of e1a2 transcripts as previously described [36,37].

### 4.3. Immunofluorescence Staining of γH2AX and 53BP1

γH2AX and 53BP1 were detected in the PBMCs of healthy donors and CML patients using a mouse monoclonal anti-γH2AX antibody (clone JBW301; Merck Millipore, Darmstadt, Germany) and a polyclonal rabbit anti-53BP1 antibody (NB100-304; Novus Biologicals, Littleton, US), respectively. An Alexa Fluor 488-conjugated goat anti-mouse antibody and an Alexa Fluor 555-conjugated goat anti-rabbit antibody (Thermo Fisher Scientific) were used as previously described [38,39]. At least 50 PBMCs were analyzed for each measurement.

### 4.4. Western Blotting

Western blotting of (p-)ATM and (p-)CHK2 was conducted in the PBMCs of healthy donors, CP-CML patients in DMR or MMR, CP-CML patients with loss of MMR, de novo untreated CP-CML patients, and BP-CML patients as previously described [39]. (p-)ATM and (p-)CHK2 expressing SKM-1 acute myeloid leukemia cells (Leibniz Institute DSMZ–German Collection of Microorganisms and Cell Cultures GmbH, Braunschweig, Germany) served as positive control.

### 4.5. Statistics

Statistical calculations were performed using SAS software, release 9.4 (SAS Institute Inc., Cary, NC, USA). Quantitative variables were stated by mean values and standard errors. The number of foci in each sample were modeled by a Poisson regression model using the SAS procedure PROC GENMOD. Furthermore, the SAS “repeated” statement was applied with statistical consideration encompassing several cells, which were counted in each sample. *p* values < 0.05 were regarded statistically significant.

## 5. Conclusions

Our study reveals accumulation of DNA damage in the course from CP-CML towards BP-CML. In addition, our data suggest increase of DNA damage, erroneous DSB repair, and alterations of the DDR as critical mediators of blastic transformation in CML. Finally, γH2AX and 53BP1 foci might be useful markers for targeting genetic instability in TKI-refractory CML in future studies.

## Figures and Tables

**Table 1:** Sample characterization of healthy donors and CML patients.

Gp	Pt	Age/Sex	Disease	TKI	γH2AX Foci Per Nucleus ± SEM	BCR/ABL MR	BCR/ABL Transcript	Cytogenetics/FISH
**Group 1**	HEALTHY#1	49/♂	Healthy	-	0.6 ± 0.1	-	-	-
	HEALTHY#2	62/♀	Healthy	-	0.7 ± 0.1	-	-	-
	HEALTHY#3	43/♂	Healthy	-	0.7 ± 0.1	-	-	-
	HEALTHY#4	NA	Healthy	-	0.9 ± 0.2	-	-	-
	HEALTHY#5	54/♀	Healthy	-	1.2 ± 0.2	-	-	-
	HEALTHY#6	36/♀	Healthy	-	1.2 ± 0.2	-	-	-
	HEALTHY#7	54/♀	Healthy	-	1.3 ± 0.1	-	-	-
	HEALTHY#8	53/♀	Healthy	-	1.6 ± 0.2	-	-	-
**Group 2**	CP-CML DMR#1	61/♀	CP-CML	DASA	0.1 ± 0.1	MR4.5	e13a2 (b2a2)	46,XX,t(9;22)(q34;q11) [25]
	CP-CML DMR#2	64/♀	CP-CML	Stop	0.4 ± 0.1	MR4.5	e13a2 (b2a2)	NA
	CP-CML DMR#3	74/♀	CP-CML	Stop	0.7 ± 0.2	MR4.5	e13a2/e14a2 (b2a2/b3a2)	NA
	CP-CML DMR#4	48/♂	CP-CML	Stop	0.7 ± 0.2	MR4.5	e14a2 (b3a2)	NA
	CP-CML DMR#5	63/♀	CP-CML	Stop	0.8 ± 0.2	MR4.5	e14a2 (b3a2)	NA
	CP-CML DMR#6	55/♂	CP-CML	DASA	0.8 ± 0.2	MR4	e14a2 (b3a2)	NA
	CP-CML DMR#7	61/♀	CP-CML	Stop	0.8 ± 0.2	MR4.5	e14a2 (b3a2)	NA
	CP-CML DMR#8	33/♀	CP-CML	DASA	0.8 ± 0.2	MR4	e13a2 (b2a2)	NA
	CP-CML DMR#9	58/♂	CP-CML	Stop	0.8 ± 0.2	MR5	e14a2 (b3a2)	NA
	CP-CML DMR#10	72/♂	CP-CML	Stop	0.9 ± 0.2	MR4.5	e14a2 (b3a2)	NA
	CP-CML DMR#11	34/♂	CP-CML	DASA	0.9 ± 0.1	MR4	e13a2 (b2a2)	NA
	CP-CML DMR#12	68/♀	CP-CML	DASA	0.9 ± 0.2	MR4	e13a2 (b2a2)	NA
	CP-CML DMR#13	78/♀	CP-CML	NILO	1.0 ± 0.2	MR4.5	e13a2/e14a2 (b2a2/b3a2)	NA
	CP-CML DMR#14	59/♀	CP-CML	IMA	1.0 ± 0.2	MR4	e13a2 (b2a2)	NA
	CP-CML DMR#15	60/♀	CP-CML	DASA	1.1 ± 0.2	MR4.5	e14a2 (b3a2)	NA
	CP-CML DMR#16	67/♀	CP-CML	Stop	1.1 ± 0.2	MR4.5	e14a2 (b3a2)	NA
	CP-CML DMR#17	76/♀	CP-CML	Stop	1.1 ± 0.2	MR4.5	e13a2 (b2a2)	NA
	CP-CML DMR#18	77/♂	CP-CML	Stop	1.2 ± 0.2	MR4.5	e13a2/e14a2 (b2a2/b3a2)	NA
	CP-CML DMR#19	84/♀	CP-CML	IMA	1.3 ± 0.2	MR4	e13a2 (b2a2)	NA
	CP-CML DMR#20	71/♀	CP-CML	Stop	1.3 ± 0.3	MR5	e14a2 (b3a2)	NA
	CP-CML DMR#21	63/♂	CP-CML	Stop	1.3 ± 0.2	MR4.5	e14a2 (b3a2)	NA
	CP-CML DMR#22	62/♂	CP-CML	DASA	1.7 ± 0.2	MR4.5	e14a2 (b3a2)	NA
	CP-CML MMR#1	70/♀	CP-CML	IMA	0.8 ± 0.2	0.04%	e13a2 (b2a2)	NA
	CP-CML MMR#2	59/♀	CP-CML	Stop	1.2 ± 0.2	0.07%	e13a2/e14a2 (b2a2/b3a2)	NA
	CP-CML MMR#3	77/♀	CP-CML	DASA	1.2 ± 0.2	0.09%	e14a2 (b3a2)	NA
	CP-CML MMR#4	54/♂	CP-CML	DASA	1.3 ± 0.2	0.02%	e14a2 (b3a2)	NA
**Group 3**	CP-CML loss MMR#1	54/♀	CP-CML	NILO	1.0 ± 0.2	0.18%	e1a2	46,XX,t(9;22)(q34;q11) [25]
	CP-CML loss MMR#2	46/♂	CP-CML	DASA	1.1 ± 0.2	45%	e14a2 (b3a2)	51,XY,+6,+8,+8,+8, t(9;22)(q34;q11),+19 [25]
	CP-CML loss MMR#3	81/♂	CP-CML	Stop	1.3 ± 0.2	0.35%	e13a2/e14a2 (b2a2/b3a2)	NA
	CP-CML loss MMR#4 *	83/♀	CP-CML	Stop	2.4 ± 0.2	1.00%	e14a2 (b3a2)	NA
	CP-CML loss MMR#5	83/♀	CP-CML	Stop	3.0 ± 0.2	30%	e13a2/e14a2 (b2a2/b3a2)	NA
**Group 4**	CP-CML de novo#1	19/♂	CP-CML	-	1.4 ± 0.2	60%	e13a2 (b2a2)	46,XY,t(9;22)(q34;q11) [25]
	CP-CML de novo#2	79/♀	CP-CML	-	1.6 ± 0.3	63%	e13a2 (b2a2)	46,XX,t(9;22)(q34;q11) [25]
	CP-CML de novo#3	18/♀	CP-CML	-	2.3 ± 0.3	68%	e13a2 (b2a2)	46,XX,t(9;22)(q34;q11) [25]
	CP-CML de novo#4	66/♀	CP-CML	-	3.4 ± 0.2	29%	e14a2 (b3a2)	46,XX,t(9;22)(q34;q11) [25]
	CP-CML de novo#5	53/♀	CP-CML	-	3.7 ± 0.3	68%	e13a2 (b2a2)	46,XX,t(9;22)(q34;q11) [25]
**Group 5**	BP-CML#1 **	76/♀	BP-CML	Stop	3.4 ± 0.4	32%	e13a2 (b2a2)	NA (rejected)
	BP-CML#2	63/♂	BP-CML	DASA	4.0 ± 0.4	63%	e14a2 (b3a2)	46,XY,inv(3)(q21;q26), t(9;22)(q34;q11) [25]
	BP-CML#3 ***	38/♀	BP-CML	PONA	5.8 ± 0.4	256%	e13a2 (b2a2)	46,XX,t(9;22)(q34;q11) [1] 44,XX,der(3)t(3;9)(p11;q11) t(9;22)(q34;q11),−7,−9, der(13)t(7;13)(q22;q34),der(22) t(9;22)(q34;q11) [5] 46,XX [14]

“Stop” = no TKI treatment. BP-CML, blast phase CML; CML, chronic myeloid leukemia; CP-CML, chronic phase CML; DASA, dasatinib; DMR, deep molecular response; FISH, fluorescence in situ hybridization; Gp, group; IMA, imatinib; MMR, major molecular response; MR, molecular response; NA, not assessed; NILO, nilotinib; PONA, ponatinib; Pt, patient; SEM, standard error of mean; TKI, tyrosine kinase inhibitor; ♀, female; ♂, male; *, V379I detected; **, M351V, E459K detected; ***, T315I detected.

## Abbreviations

AP	Accelerated phase
ACA	Additional chromosomal aberrations
BP	Blast phase
CML	Chronic myeloid leukemia
CP	Chronic phase
DASA	Dasatinib
DDR	DNA damage response
DMR	Deep molecular response
DSB	DNA double-strand breaks
IMA	Imatinib
MMEJ	Microhomology mediated end-joining
MMR	Major molecular response
MR	Molecular response
NHEJ	Non-homologous end joining
NILO	Nilotinib
PBMC	Peripheral blood mononuclear cells
PONA	Ponatinib
ROS	Reactive oxygen species
TKI	Tyrosine kinase inhibitor

## References

[1] Vardiman J.W., Melo J.V., Baccarani M., Radich J.P., Kvasnicka H.M., Swerdlow S.H., Campo E., Harris N.L., Jaffe E.S., Pileri S.A., Stein H., Thiele J., Arber D.A., Hasserjian R.P., Le Beau M.M. (2017). Chronic myeloid leukaemia, BCR-ABL1-positive. WHO Classification of Tumours of Haematopoietic and Lymphoid Tissues.

[2] NCCN Chronic Myeloid Leukemia, Version 1.2019, NCCN Clinical Practice Guidelines in Oncology. https://jnccn.org/view/journals/jnccn/16/9/article-p1108.xml.

[3] Holyoake T.L., Vetrie D. (2017). The chronic myeloid leukemia stem cell: Stemming the tide of persistence. Blood.

[4] Muvarak N., Nagaria P., Rassool F.V. (2012). Genomic instability in chronic myeloid leukemia: Targets for therapy?. Curr. Hematol. Malig. Rep..

[5] Nowicki M.O., Falinski R., Koptyra M., Slupianek A., Stoklosa T., Gloc E., Nieborowska-Skorska M., Blasiak J., Skorski T. (2004). BCR/ABL oncogenic kinase promotes unfaithful repair of the reactive oxygen species-dependent DNA double-strand breaks. Blood.

[6] Koptyra M., Cramer K., Slupianek A., Richardson C., Skorski T. (2008). BCR/ABL promotes accumulation of chromosomal aberrations induced by oxidative and genotoxic stress. Leukemia.

[7] Cramer K., Nieborowska-Skorska M., Koptyra M., Slupianek A., Penserga E.T., Eaves C.J., Aulitzky W., Skorski T. (2008). BCR/ABL and other kinases from chronic myeloproliferative disorders stimulate single-strand annealing, an unfaithful DNA double-strand break repair. Cancer Res..

[8] Stoklosa T., Poplawski T., Koptyra M., Nieborowska-Skorska M., Basak G., Slupianek A., Rayevskaya M., Seferynska I., Herrera L., Blasiak J. (2008). BCR/ABL inhibits mismatch repair to protect from apoptosis and induce point mutations. Cancer Res..

[9] Baccarani M., Deininger M.W., Rosti G., Hochhaus A., Soverini S., Apperley J.F., Cervantes F., Clark R.E., Cortes J.E., Guilhot F. (2013). European LeukemiaNet recommendations for the management of chronic myeloid leukemia: 2013. Blood.

[10] Patel A.B., O’Hare T., Deininger M.W. (2017). Mechanisms of Resistance to ABL Kinase Inhibition in Chronic Myeloid Leukemia and the Development of Next Generation ABL Kinase Inhibitors. Hematol. Oncol. Clin. North. Am..

[11] Rogakou E.P., Pilch D.R., Orr A.H., Ivanova V.S., Bonner W.M. (1998). DNA double-stranded breaks induce histone H2AX phosphorylation on serine 139. J. Biol. Chem..

[12] Scully R., Xie A. (2013). Double strand break repair functions of histone H2AX. Mutat Res..

[13] Stucki M., Clapperton J.A., Mohammad D., Yaffe M.B., Smerdon S.J., Jackson S.P. (2005). MDC1 directly binds phosphorylated histone H2AX to regulate cellular responses to DNA double-strand breaks. Cell.

[14] Kobayashi J., Tauchi H., Sakamoto S., Nakamura A., Morishima K., Matsuura S., Kobayashi T., Tamai K., Tanimoto K., Komatsu K. (2002). NBS1 localizes to gamma-H2AX foci through interaction with the FHA/BRCT domain. Curr. Biol..

[15] Paull T.T., Rogakou E.P., Yamazaki V., Kirchgessner C.U., Gellert M., Bonner W.M. (2000). A critical role for histone H2AX in recruitment of repair factors to nuclear foci after DNA damage. Curr. Biol..

[16] Ward I.M., Minn K., Jorda K.G., Chen J. (2003). Accumulation of checkpoint protein 53BP1 at DNA breaks involves its binding to phosphorylated histone H2AX. J. Biol. Chem..

[17] Difilippantonio S., Gapud E., Wong N., Huang C.Y., Mahowald G., Chen H.T., Kruhlak M.J., Callen E., Livak F., Nussenzweig M.C. (2008). 53BP1 facilitates long-range DNA end-joining during V(D)J recombination. Nature.

[18] Lee J.H., Paull T.T. (2007). Activation and regulation of ATM kinase activity in response to DNA double-strand breaks. Oncogene.

[19] Xiong X., Du Z., Wang Y., Feng Z., Fan P., Yan C., Willers H., Zhang J. (2015). 53BP1 promotes microhomology-mediated end-joining in G1-phase cells. Nucleic Acids Res..

[20] Callen E., Di Virgilio M., Kruhlak M.J., Nieto-Soler M., Wong N., Chen H.T., Faryabi R.B., Polato F., Santos M., Starnes L.M. (2013). 53BP1 mediates productive and mutagenic DNA repair through distinct phosphoprotein interactions. Cell.

[21] Chapman J.R., Barral P., Vannier J.B., Borel V., Steger M., Tomas-Loba A., Sartori A.A., Adams I.R., Batista F.D., Boulton S.J. (2013). RIF1 is essential for 53BP1-dependent nonhomologous end joining and suppression of DNA double-strand break resection. Mol. Cell.

[22] Bartkova J., Horejsi Z., Koed K., Kramer A., Tort F., Zieger K., Guldberg P., Sehested M., Nesland J.M., Lukas C. (2005). DNA damage response as a candidate anti-cancer barrier in early human tumorigenesis. Nature.

[23] Norbury C.J., Zhivotovsky B. (2004). DNA damage-induced apoptosis. Oncogene.

[24] Roos W.P., Kaina B. (2013). DNA damage-induced cell death: From specific DNA lesions to the DNA damage response and apoptosis. Cancer Lett..

[25] Roos W.P., Thomas A.D., Kaina B. (2016). DNA damage and the balance between survival and death in cancer biology. Nat. Rev. Cancer.

[26] Mitelman F. (1993). The cytogenetic scenario of chronic myeloid leukemia. Leuk Lymphoma.

[27] Fabarius A., Leitner A., Hochhaus A., Muller M.C., Hanfstein B., Haferlach C., Gohring G., Schlegelberger B., Jotterand M., Reiter A. (2011). Impact of additional cytogenetic aberrations at diagnosis on prognosis of CML: Long-term observation of 1151 patients from the randomized CML Study IV. Blood.

[28] Vilenchik M.M., Knudson A.G. (2003). Endogenous DNA double-strand breaks: Production, fidelity of repair, and induction of cancer. Proc. Natl. Acad. Sci. USA.

[29] Bunting S.F., Nussenzweig A. (2013). End-joining, translocations and cancer. Nat. Rev. Cancer.

[30] Halazonetis T.D., Gorgoulis V.G., Bartek J. (2008). An oncogene-induced DNA damage model for cancer development. Science.

[31] Tobin L.A., Robert C., Rapoport A.P., Gojo I., Baer M.R., Tomkinson A.E., Rassool F.V. (2013). Targeting abnormal DNA double-strand break repair in tyrosine kinase inhibitor-resistant chronic myeloid leukemias. Oncogene.

[32] Löffler H., Rastetter J., Haferlach T. (2005). Light microscopic procedures. Atlas of Clinical Hematology.

[33] MLL Request for Testing Form. https://www.mll.com/en.html.

[34] Gisselsson D., Heim S., Mitelman F. (2009). Cytogenetic methods. Cancer Cytogenetics.

[35] UMM Request for Testing Form. https://www.umm.de/iii-medizinische-klinik/wissenschaftliches-labor/.

[36] Emig M., Saussele S., Wittor H., Weisser A., Reiter A., Willer A., Berger U., Hehlmann R., Cross N.C., Hochhaus A. (1999). Accurate and rapid analysis of residual disease in patients with CML using specific fluorescent hybridization probes for real time quantitative RT-PCR. Leukemia.

[37] Spiess B., Rinaldetti S., Naumann N., Galuschek N., Kossak-Roth U., Wuchter P., Tarnopolscaia I., Rose D., Voskanyan A., Fabarius A. (2019). Diagnostic performance of the molecular BCR-ABL1 monitoring system may impact on inclusion of CML patients in stopping trials. PLoS ONE.

[38] Popp H.D., Brendel S., Hofmann W.K., Fabarius A. (2017). Immunofluorescence Microscopy of gammaH2AX and 53BP1 for Analyzing the Formation and Repair of DNA Double-strand Breaks. J. Vis. Exp..

[39] Popp H.D., Naumann N., Brendel S., Henzler T., Weiss C., Hofmann W.K., Fabarius A. (2017). Increase of DNA damage and alteration of the DNA damage response in myelodysplastic syndromes and acute myeloid leukemias. Leuk Res..

